# The Vitamin K Metabolome in Chronic Kidney Disease

**DOI:** 10.3390/nu10081076

**Published:** 2018-08-12

**Authors:** Mandy E. Turner, Michael A. Adams, Rachel M. Holden

**Affiliations:** 1 Department of Biomedical and Molecular Science, Queen’s University, Kingston, ON K7L 3V6, Canada; mandy.turner@queensu.ca (M.E.T.); adams@queensu.ca (M.A.A.); 2 Department of Medicine, Queen’s University, Kingston, ON K7L 3V6, Canada

**Keywords:** vitamin K, chronic kidney disease, phylloquinone, menaquinone-4, vitamin K dependent proteins

## Abstract

The purpose of this review is to summarize the research to date on the impact of chronic kidney disease (CKD) on the vitamin K metabolome. Vitamin K-dependent proteins contribute to cardiovascular disease (CVD) prevention via the prevention of ectopic mineralization. Sub-clinical vitamin K deficiency is common in CKD patients, and evidence suggests that it may contribute to the CVD burden in this population. Research from animal models suggests that CKD alters tissue measures of the two predominant forms of vitamin K: K_I_ and MK-4. The expression and/or activity of enzymes that regulate the recycling of vitamin K and the carboxylation of vitamin K-dependent proteins also appear to be altered in CKD. Evidence suggests that statins, a common pharmaceutical prescribed to CKD patients to prevent cardiovascular events, may impact the metabolism of vitamin K and therefore contribute to its relative inefficiency at preventing CVD in this population as kidney disease progresses. Human research on the tissue vitamin K metabolome in CKD patients is lacking.

## 1. Introduction

Chronic kidney disease (CKD) affects approximately 14% of the adult population in the United States, where it aligns closely with the prevalence of diabetes and hypertension [1]. Low kidney function is linked to poor health outcomes overall and specifically a much higher risk of cardiovascular disease [2]. The risk of cardiovascular disease exponentially increases as kidney function declines [2]. This is due in part to vascular calcification, which is a sequela of the bone and mineral disorder that accompanies low kidney function.

CKD patients have a very high prevalence of sub-clinical vitamin K deficiency that is characterized by low levels of circulating vitamin K and high levels of inactive vitamin K-dependent proteins (VKDPs) in the circulation [3,4,5,6,7]. Vitamin K is an umbrella term for a group of fat-soluble vitamins that are implicated in bone and vascular health. The two major forms of vitamin K include phylloquinone, or vitamin K_1_, and menaquinones, which are collectively referred to as vitamin K_2_. Both vitamin K forms can function as a co-factor for the enzyme α-glutamyl carboxylase (GGCX), which performs the critical step in the activation of VKDPs.

As kidney function declines, phosphate retention occurs. Phosphate is a key signaling molecule mediating the conversion of vascular smooth muscle cells to osteoblast-like cells that produce bone matrix proteins that mediate mineralization [8]. Two vitamin K-dependent proteins have significant roles in the mineralization process. Osteocalcin is a VKDP that is produced by mature osteoblasts and mediates bone mineralization and anabolism. Matrix gla protein (MGP) is a VKDP that is expressed by vascular smooth muscle and endothelial cells and inhibits soft tissue mineralization. MGP becomes highly upregulated adjacent to sites of arterial calcification and is thought to inhibit vascular calcification indirectly by interfering with BMP-2 function or by directly binding extracellular calcium [9]. Both osteocalcin and MGP require vitamin K-mediated gamma-carboxylation in order to be active. In the general population, high levels of inactive osteocalcin and MGP in the circulation have been linked to osteoporosis and cardiovascular disease, respectively [10,11]. Patients with CKD consistently have been identified to be a group with very high levels of inactive osteocalcin and MGP [3,4,5,6,7]. The level of the circulating inactive form of MGP appears to reflect vitamin K status and has been linked to vascular calcification in CKD patients and with the deterioration of kidney function in the general population [12,13].

There is growing evidence to suggest that the uremic milieu contributes to abnormalities in the overall vitamin K metabolome. Thus, patients with CKD may be at unique risk for biological consequences related to vitamin K deficiency. The purpose of this review is to discuss what is known about the vitamin K metabolome in the setting of CKD and the potential impact of pharmacological interventions on vitamin K metabolism in this population.

## 2. Assessment of Vitamin K Adequacy in CKD Patients

There is presently no gold standard measure to assess vitamin K adequacy in humans; therefore, a combination of dietary intake measures and biomarkers are used. Phylloquinone (vitamin K_1_) is the major dietary source of vitamin K and is found in green leafy vegetables and vegetable oils. Menaquinones (vitamin K_2_) are derived from highly fermented foods and animal-based food products such as dairy and meat. Menaquinones make up a relatively low percentage of the total dietary vitamin K intake in a typical western diet. The recommended adequate intake (AI) of vitamin K is 120 and 90 μg/day for men and women, respectively [14]. This value is recommended by the United States (US) Institute of Medicine and is based on population-based intake studies and the maintenance of adequate coagulation. There are no AI guidelines for menaquinones, despite being re-evaluated recently by the European Food Safety Authority. They contend that the available evidence on menaquinone intake, absorption, function, and tissue distribution is not sufficient at present to warrant separate AI guidelines [15]. Non-coagulation-based vitamin K requirements have not been assessed and could be different. 

Diets rich in vitamin K_1_ and vitamin K_2_ are also often high in potassium and phosphorus, respectively, both of which must be restricted in CKD patients. Thus, it has been suggested that vitamin K deficiency in CKD patients may be due, in part, to a low overall intake of vitamin K. Several studies have been conducted to assess the vitamin K intake of patients with low kidney function. Cranenburg et al. conducted 4-day diet records in Dutch hemodialysis patients and reported that the median overall intake of vitamin K_1_ was 118 μg/day in the group, thus appearing quite similar to the AI recommended for the general population [3]. Vitamin K_1_ intake was assessed in Polish hemodialysis patients and compared with a healthy control group [16]. Overall, vitamin K intake was similar between the groups but was below the recommended AI for vitamin K_1_. In contrast, vitamin K_1_ intake was significantly lower in Italian hemodialysis patients compared with healthy people, although the median intake in both groups in this study exceeded the recommended AI [17]. Although there are no recommendations for vitamin K_2_ intake, hemodialysis patients consumed significantly less vitamin K_2_ than did their healthy counterparts in the two studies where it has been addressed [3,16]. Patients with earlier stages of CKD reported intakes of vitamin K_1_ that aligned well with recommended AI amounts [5]. Taken together, the vitamin K intake of dialysis patients may be lower than clinical reference groups but are not consistently below the AI recommendations.

## 3. Circulating and Tissue Measures of Vitamin K_1_ and Vitamin K_2_ in CKD

Phylloquinone (vitamin K_1_) is the form of vitamin K measured in the circulation of humans and rodents fed a phylloquinone-containing purified diet. Classically, the level of phylloquinone in the blood is felt to best reflect tissue stores and thus globally assesses the combination of nutrient intake, absorption, and metabolism [18]. Studies consistently report that hemodialysis patients have low circulating concentrations of vitamin K_1_ [3,4,6]. Between 30% and 50% of hemodialysis patients in these studies demonstrated values below the lower limit of the normal range (<0.4 nmol/L), whilst in a study of earlier stage CKD patients, only 6% of patients were deficient [5]. MK-4 is typically not detected in the serum at high concentrations and was not detected at all in the Dutch hemodialysis patients [3]. In contrast, the Polish group detected MK-4 in 59% of their hemodialysis patients (compared with 95% of control subjects), but the values were significantly lower in the hemodialysis patients compared with the healthy reference group [16]. Both studies used high-performance liquid chromatography-based analysis. It should be noted as a limitation that blood menaquinone measurements are not subjected to the same quality assurance protocols as clinical measurement of phylloquinones and are thus less reliable.

Different forms of vitamin K predominate in different tissues, and there is considerable evidence supporting endogenous conversion between vitamin K types through a menadione intermediate. Vitamin K_1_ is the predominant form of vitamin K that is measured in plasma, the liver, and the heart. Conversely, high levels of MK-4 are found in certain tissues, such as brain, kidney, and pancreas, and very low levels are found in plasma and the liver. Some tissues appear to regulate their MK-4 concentrations (e.g., brain, reproductive organs, fat) whilst others appear to accumulate MK-4 according to the dietary level of K_1_ [19]. This tissue-specific distribution suggests unique functions for vitamin K, specifically MK-4, that are not currently appreciated. UbiA prenltransferase containing 1 (UBIAD1) has been identified as one biosynthetic enzyme involved in the conversion of menadione to MK-4 in certain tissues [20]. There are few data available that address the regulation of UBIAD1.

To the best of our knowledge, tissue levels of K_1_ and MK-4 have not been measured in patients with chronic kidney disease. In a rat model of experimental CKD, significant differences with respect to the concentrations of K vitamins in tissues were observed [21,22]. Significantly lower levels of vitamin K_1_ were detected in the liver and heart, and higher levels of MK-4 were detected in the kidneys of CKD animals compared with the controls. These differences were evident at 3 weeks of CKD, a timepoint when creatinine and phosphate were only mildly elevated compared with healthy rats, and no vascular calcification was present [21]. Thus, there appears to be an early impact of uremia on vitamin K metabolism in some tissues. The tissue-specific distribution of vitamin K forms in CKD did not differ from that in healthy rats. Although human studies consistently report low serum levels of vitamin K_1_ in dialysis patients, levels in CKD rats were non-significantly higher than the control rats [21]. The reason for this difference is not clear but indicates possible species-specific factors. To the best of our knowledge, no other study has measured vitamin K_1_ serum levels in an experimental CKD model.

Important to consider in the interpretation of the above data are notable sex and age differences in tissue vitamin K levels that could have significant implications when supplementing patients with CKD [23,24]. Harshman et al. characterized the sex differences in the vitamin K metabolome in C57BI6 mice exposed to vitamin K-sufficient and -deficient diets [23]. The metabolome was characterized in the liver, kidney, the brain, mesenteric adipose tissue, and the pancreas. There were significant sex-specific differences in all tissues for measurements of K_1_, MK-4, and various other long-chain MKs. Female rats, while not having higher circulating K_1_, had significantly higher levels of K_1_ and MK-4 in all tissues. This effect was less pronounced in rats fed the deficient diet and was not necessarily the case for the longer chain MKs. To the best of our knowledge, the impact of sex on vitamin K tissue measures has not been addressed in experimental CKD.

## 4. The Vitamin K Cycle in CKD

The α-glutamyl carboxylase (GGCX) is the protein that carboxylates and thus actives VKDPs. This reaction utilizes a reduced hydroquinone form of the vitamin K molecule, which is transformed into an epoxide during the reaction. Before the vitamin K molecule can be re-used to activate another VKDP, it must be recycled back to the reduced hydroquinone in a two-step process. First, it is transformed into a quinone (the dietary form of vitamin K) by the vitamin K epoxide reductase (VKOR), the primary target of warfarin. Less is understood about the second step, which is an additional reduction to the hydroquinone form. It is thought it may be partially mediated through VKOR, although not entirely. DT diaphorase has been identified to catalyze this reaction, although this enzyme appears only to have affinity for menadione, and knock-out experiments in mice indicate a minor role for this protein in the overall metabolic profile. Therefore, it is believed that this second step may be a result of various reduction pathways. To the best of our knowledge, only two papers have reviewed the impact of CKD on aspects of tissue vitamin K recycling, both in experimental rat models.

In experimental CKD, the development of uremia appears to alter mRNA transcript levels of VKOR; however, this does not necessarily translate to alterations in its activity. McCabe et al. reported that the kidney expression of *Vkorc1* was decreased in mild and severe CKD and that it was not rescued by supplementation with high doses of vitamin K_1_ [22]. Aortic expression of *Vkorc1* also decreased with increasing CKD severity, but the response to vitamin K supplementation was not assessed. Liver expression was unchanged. However, Kaesler et al. used an activity assay, rather than transcript levels, in the same CKD animal model and reported no change in VKOR activity in kidney or liver tissue at two levels of CKD severity or with various levels of vitamin K_1_ and vitamin K_2_ supplementation [25]. Aortic activity was not measured in this paper. The assay used quantified the tissues’ abilities to convert the vitamin K1 epoxide to the corresponding K1 quinione. While both studies above used male rats, it is important to note that sex-specific differences have been demonstrated in *Vkorc1* expression in the liver and pancreas in healthy rats [23]. In addition, in the low dietary vitamin K_1_ condition of this study, there was a suppression and elevation of *Vkorc1* in the liver and adipose tissue, respectively. This finding suggests the impact of dietary vitamin K on the regulation of recycling may be tissue dependent.

GGCX is often reported as the rate-limiting step of vitamin K recycling in CKD and Kaesler et al. suggested that its downregulation may be a potential contributor to vascular calcification [25]. Both studies of experimental CKD report no change in the liver expression of GGBX. McCabe et al. reported no change (non-significant reduction) in its expression in either the aorta or kidney [22]. However, Kaesler et al. assessed GGCX activity and reported that its activity was highly influenced by CKD and also by K supplementation, suggesting substantial post-translational regulation [25]. CKD induced a consistent (although sometimes non-significant) suppression of GGCX activity in the kidney, liver (in mild and severe CKD), and aorta (severe CKD). The effect was more pronounced in severe CKD and, in each case, was rescued by K supplementation with either K_1_ or MK-4. The expression of GGCX is regulated, in part, as a result of sex and dietary restriction. Notably, a reduced expression as a result of dietary K restriction in the liver and kidney was more apparent in male rats, and a substantial increase in brain expression as a result of dietary restriction was more apparent in female rats [23]. Whether this confers an alteration in protein expression or activity has yet to be determined.

Kaesler et al. also measured DT-diaphorase activity in the kidney and liver and reported that it was significantly increased in mild CKD; however, this effect was attenuated as CKD severity progressed [25]. The effect of vitamin K supplementation was not consistent between tissues, and aortic activity was not measured. There was an inverse correlation between GGCX activity and DT-diaphorase activity. However, in peripheral blood mononuclear cells from hemodialysis patients, the expression of DT diaphorase was significantly reduced, along with inducer, Nrf2, expression [26].

Taken together, CKD may influence vitamin K recycling in a tissue-specific manner. These primary studies also suggest that supplementation of vitamin K has the potential to normalize some elements of the metabolome. In addition, vitamin K metabolism regulation by uremia seems to be predominately post-transcriptional modification-based, and tissue RNA expression of classical enzymes often conflicts with tissue activity levels of enzyme conversion. It is a major limitation that this has only been studied in one experimental model of CKD.

## 5. Carboxylation Status of Vitamin K-Dependent Proteins

Vitamin K-dependent protein function is critical to blood coagulation, as well as bone and blood vessel health. Studies have consistently demonstrated that patients with CKD exhibit very high levels of under-carboxylated, and therefore inactive, VKDPs in the circulation. The level of under-carboxylated prothrombin (PIVKA-II), carboxylated in the liver, increases in response to a reduction in dietary vitamin K and is a measure of hepatic vitamin K status. PIVKA levels exceeded the upper range of normal (2 nmol/L) in 97% of early-stage CKD patients where it correlated weakly with dietary vitamin K intake [5]. A similar proportion of hemodialysis patients had elevated levels of PIVKA in three separate studies that utilized the same assay [3,4,7]. In contrast, PIVKA levels were similar to healthy controls in one report of Polish hemodialysis patients [27]. This discrepancy between the latter study and the previous ones could be attributed to differences in the assay used.

Patients with chronic kidney disease also demonstrate significantly increased levels of undercarboxylated osteocalcin (ucOC) levels compared with healthy controls [3,4,6,7,28]. Osteocalcin (OC) is synthesized by osteoblasts during bone formation. After it is produced, it undergoes post-synthetic modification by vitamin K-dependent carboxylation of three glutamic acid residues to convert ucOC to carboxylated osteocalcin (cOC). The cOC form is capable of binding to hydroxyapatite crystals in bone to promote bone formation. In health, the proportion of ucOC relative to total OC in the circulation typically does not exceed 20%. In contrast, levels of total OC are markedly elevated in CKD where it circulates predominantly in the ucOC form [3,4,6,7,28]. In CKD patients, the level of OC typically correlates very strongly with other biomarkers of the chronic kidney disease-mineral bone disorder (CKD-MBD) including phosphate and parathyroid hormone [6]. Further, retention of OC fragments may occur with impaired excretion into the urine as a direct result of declining kidney function. These changes in the OC axis have been observed early on in the course of CKD where a high percentage of ucOC in the circulation has been observed in 60–70% of pre-dialysis patients [5,28]. These data put into question the utility of using the percentage of OC in the under-carboxylated form as a biomarker of vitamin K status in CKD patients, the very high levels notwithstanding. 

The matrix Gla protein is a VKDP synthesized by vascular smooth muscle cells and endothelial cells. Abnormalities in its function have been linked to vascular calcification and heart disease [12,29]. A combination of vitamin K-dependent carboxylation and serine phosphorylation are required to generate active MGP. Once activated, MGP is a potent inhibitor of vascular calcification. Several studies have confirmed a 4–5-fold increase in the level of uc-dp-MGP in the circulation of hemodialysis patients and a progressive increase across stages of CKD [12]. However, the use of uc-dp-MGP as a biomarker of vitamin K status is complicated by the potential impact of CKD on increasing MGP synthesis. While the significance of MGP in the circulation is uncertain, the levels of the uc-dp-MGP have been shown to respond to dietary supplementation in hemodialysis patients [7].

Taken together, patients with chronic kidney disease appear to consume an adequate amount of vitamin K yet exhibit very high levels of under-carboxylated VKDPs in the circulation. In the general population, the under-carboxylated fraction of a VKDP is thought to reflect deficiency at the site of production of that protein. It is not clear, and unlikely, that a similar relationship exists in CKD patients. We propose that there is growing evidence to suggest that a defect in the carboxylation process contributes to the accumulation of under-carboxylated VKDPs and reflects an overall impact of uremia on vitamin K metabolism.

## 6. Overcoming Carboxylation Defects in CKD

There are published and unpublished studies to indicate that the apparent carboxylation defect in hemodialysis patients can be corrected with both vitamin K_1_ and vitamin K_2_ supplementation. There was a dose-dependent reduction of various uncarboxylated VKDPs, ucOC, uc-dp-MGP, and PIVKA, with three levels of MK-4 supplementation in a study of German dialysis patients [7]. Similarly, in a small randomized placebo-controlled cross-over study of 20 hemodialysis patients, the provision of 1 mg of vitamin K1 daily normalized PIVKA levels in 100% of hemodialysis patients (unpublished observation). These results are consistent with the one pre-clinical study that demonstrated that vitamin K normalized GGCX activity [25]. Given that the provision of vitamin K appears to correct the carboxylation status of VKDPs in hemodialysis patients, it is plausible that vitamin K supplementation could modify clinical events including cardiovascular disease and bone fracture in this patient population. Clinical trials to date have largely been performed in the general population and specifically not in CKD patients where vitamin K deficiency and vascular calcification are highly prevalent and frequently co-exist. Currently, there are two randomized controlled trials being performed in Europe and in Canada to address this question. Both studies are evaluating the impact of vitamin K_1_ supplementation on the progression of coronary artery calcification. The Canadian iPACK-HD trial is evaluating the impact of 10 mg of vitamin K_1_ over 12 months, whilst the European Vita-VasK trial is evaluating the impact of 5 mg of vitamin K_1_ over 18 months [30,31]. Both studies are being conducted in hemodialysis patients.

## 7. Pharmacological Impact on Tissue Vitamin K Metabolism: Highlight on Statin Therapy

Statins are a lipid-lowering class of drugs that are frequently prescribed to reduce cardiovascular events and mortality in the general population. Despite similar cholesterol lowering, the effectiveness of statin treatment is attenuated as kidney disease progresses with some evidence of harm in dialysis patients as demonstrated by an increased risk of stroke in the 4D trial [32]. Recent evidence supports the hypothesis that statin therapy may alter the vitamin K metabolome; specifically, there is animal model evidence supporting the inhibition of the tissue generation of MK-4 [33].

Statins inhibit HMG-CoA reductase, a key enzyme involved in endogenous cholesterol synthesis. Geranylgeranyl pyrophosphate, a molecule upstream from HMG-CoA in the cholesterol synthesis pathway, donates a geranylgeranyl (GGpp) moiety that is used by UBIAD1 to synthesize MK-4 from menadione [34]. In addition, GGpp regulates intracellular trafficking of UBIAD1 [34]. One hypothesis, therefore, is that in the presence of a statin, the supply of GGpp to UBIAD1 may be reduced in favor of directing this substrate towards cholesterol synthesis. The impact of statins on the vitamin K metabolome was recently demonstrated in a study of healthy mice fed a vitamin K_1_ diet for 7 weeks, with one group of mice concurrently receiving atorvastatin therapy [33]. During the 8th week, the animals received vitamin K_1_ that was labeled with deuterium. The mice receiving statin therapy had a 41% and 47% reduction in MK-4 and labeled MK-4, respectively, in kidney tissue. No differences were found in the liver where MK-4 is typically not generated. Further, no differences were found in brain levels of MK-4 or labeled MK-4. It was suggested that this may be a consequence of atorvastatin not being able to cross the blood–brain barrier. This is one of the first studies to demonstrate an impact of statin therapy on tissue generation of MK-4. Many hemodialysis patients continue to receive statins despite KDIGO recommendations that they are not clearly indicated in this group. In a recent study, hemodialysis patients taking statins had higher baseline coronary artery calcification and greater progression of calcification over a median of 1.5 years of follow-up [35]. Whilst worsening sub-clinical vitamin K deficiency in the presence of a statin is an attractive hypothesis, one cannot rule out the very distinct possibility of bias by indication. Taken together, further research is required, particularly in earlier stages of CKD where statin treatment has been shown to reduce clinical events and its use is widely supported by clinical practice guidelines [36].

## 8. Conclusions

In this review, we have highlighted that patients with CKD are a clinical group at high risk for low circulating levels of vitamin K_1_ and elevated levels of dysfunctional VKDPs. Pre-clinical studies indicate an impact of uremia on tissue levels of vitamin K and on the activity of GGCX, the key enzyme involved in activation of VKDPs. These findings suggest a direct impact of CKD on vitamin K recycling and the carboxylation process. In experimental uremia, both are amenable to vitamin K treatment. Small trials in patients with end-stage kidney disease requiring dialysis have similarly demonstrated that the carboxylation of VKDPs is responsive to vitamin K treatment. Whether vitamin K treatment will have an impact upon the downstream targets of VKDPs, including the protection of the vasculature and bone, is unknown but currently being studied in clinical trials.
